# Biomarkers of Inflammation among Patients with COVID-19: A Single-Centre Prospective Study from Prishtina, Kosovo

**DOI:** 10.1155/2022/4461647

**Published:** 2022-07-15

**Authors:** Hatixhe Latifi-Pupovci, Sadie Namani, Blerina Ahmetaj-Shala, Artina Pajaziti, Gramoz Bunjaku, Lindita Ajazaj Berisha, Valentin Gegaj, Afrim Kotori

**Affiliations:** ^1^University of Prishtina, Prishtina, Kosovo; ^2^University Clinical Center of Kosovo, Prishtina, Kosovo; ^3^National Heart and Lung Institute, Imperial College, London, UK; ^4^University for Business and Technology, Prishtina, Kosovo

## Abstract

SARS-CoV-2 infection involves the phase of viral replication and inflammatory response predicting the severity of COVID-19. The aim of the study was to analyze the association between IL-6 and hematological and inflammatory parameters and outcomes of patients with COVID-19. Plasma interleukin 6 (IL-6) levels and other inflammatory and hematological parameters were analyzed in 86 adult patients diagnosed with SARS-CoV-2 infection in Kosovo. The median age of patients was 61.50 (49.75–67.25) years. Over half of patients were categorised as severe (58%) and had comorbidities (69%) with hypertension being the most common. The overall mortality rate was 4.7%. The distribution of biochemical parameters across disease severity groups was significantly different for C-reactive protein (CRP), lactate dehydrogenase (LDH), erythrocyte sedimentation rate (ESR), white blood cells (WBC), and granulocytes with higher median values in more severe and critically ill patients whereas lower percentage of lymphocytes, monocytes, and platelet count in severe and critically ill patients. IL-6 levels were increased in 63% of patients with significant differences in the distribution across the following groups; age, disease severity, hospitalisation status, pulmonary infiltrates, oxygen therapy, and hypertension status. IL-6 significantly correlated with CRP, LDH, CK, ESR, and percentages of granulocytes. IL-6 and other inflammatory and hematological parameters were strongly associated with disease severity and may predict the outcome of the SARS-CoV-2 infection.

## 1. Introduction

On March 11, 2020, the World Health Organization (WHO) declared the novel coronavirus (COVID-19) outbreak a global pandemic. To date (May 4th, 2022), there have been 515.82 million confirmed cases of COVID-19 worldwide, with 6.24 million reported deaths; whereas in Kosovo, 228,098 cases were confirmed and 3,138 deaths reported [1].

In patients, COVID-19 results in a wide range of clinical manifestations, including asymptomatic infection, mild upper respiratory syndrome, severe pneumonia, acute respiratory distress syndrome (ARDS), multiple organ dysfunction syndrome, and even death. The most prevalent symptoms are fever, cough [2, 3], headache, loss of smell, and nasal obstruction [4], whereas gastrointestinal disorders are uncommon [2, 3]. The risk of severe disease and death increases with age and the presence of comorbidities such as cardiovascular diseases and diabetes mellitus [5, 6]. The reason for increased disease severity and even death in some patients remains unclear, especially in young adults with no comorbidities. Indeed, hyperinflammatory responses induced by SARS-CoV-2 combined with bacterial infection remain one of the major causes of disease severity and death in adults [7, 8].

Various inflammatory markers were associated with COVID-19 progression [8], but the hallmark of hyperinflammatory response is interleukin-6 (IL-6) acting as a central player in immune regulation, inflammation, and infection [9]. Raised levels of IL-6 precede the development of acute lung injury due to increased permeability of lung capillaries driving the ARDS development and stimulation of coagulation and microthrombi formation in the lungs [10]. Patients with higher IL-6 levels have more rapid progression and a higher complication rate so it was proposed that the levels of IL-6 may be used as a biomarker to help assist clinicians in recognizing patients with severe COVID‐19 early in the disease course [11, 12]. The importance of identifying this elevated biomarker also lies in selecting patients who can benefit from the use of antibodies against IL-6 such as tocilizumab. IL-6 also induces the synthesis of another sensitive biomarker of inflammation and infection, CRP, which increases during inflammatory response [13]. Due to the fact that it is very critical to identify patients with high chances of worsening clinical symptoms or poor clinical outcomes, it is very helpful to find laboratory parameters, which can predict disease worsening. Therefore, the aim of this study was to analyze the association between IL-6 and hematological and inflammatory parameters with outcomes of patients with COVID-19 in Kosovo.

## 2. Material and Methods

### 2.1. Study Group

In this prospective study, a total of 86 COVID-19 patients diagnosed by RT-PCR test following a nasopharyngeal swab were included. Of these, 73 were recruited when hospitalized in the Infectious Diseases Clinic and the Pulmonology Clinic at the University Clinical Center of Kosovo and 13 were seen as outpatients. Fifty patients in this study group are also part of the project “Relationship of anti-SARS-CoV-2 IgG antibodies with Vitamin D and inflammatory markers in COVID-19 patients.” All blood samples were obtained at the time of admission to the hospital whereas demographic, clinical, and laboratory data were obtained from the medical records of patients. Laboratory data of ambulatory patients were collected during specialist visits.

The clinical staging of patients was generally categorised into 4 groups based on the COVID-19 clinical management guideline issued by WHO [14]: mild, moderate, severe, and critical. Mild cases: 9 (10.5%) patients with symptoms of COVID-19 but without signs of hypoxia and pneumonia on imaging; moderate illness: 23 (26.7%) patients with fever, cough, dyspnea, rapid breathing, SpO_2_> 93%, and pneumonia on imaging; severe illness: 50 (58.1%) patients with clinical signs of pneumonia (fever, cough, dyspnea and one of these signs: respiratory rate> 30/min, severe respiratory distress, or SpO_2_ <93% on room air); critical illness: 4 (4.7%) patients with acute respiratory distress syndrome (ARDS)—bilateral lung opacities and oxygenation disorder, respiratory failure, septic shock, and/or multiple organ dysfunction.

This study was approved by the Ethics Committee at the University Clinical Center of Kosovo (Reference number 1009). All patients gave written informed consent prior to inclusion in the study in accordance with the Declaration of Helsinki.

### 2.2. Inclusion and Exclusion Criteria

Adults of all ethnicities, ages, and genders were eligible to participate in the study. Study exclusion criteria included refusal to participate in the study, refusal to provide informed consent, or a medical contraindication to blood donation (e.g., severe anaemia) and pregnant women.

### 2.3. IL-6 Quantification

For IL-6 quantification, 4 ml of blood was drawn from the cubital vein using granules and clot activator tubes. Serum was separated after centrifugation at 3500 rpm for 10 min and was stored in a refrigerator at −80°C until further analysis. IL-6 levels were measured by the electrochemiluminescence immunoassay (ECLIA) using the Elecsys kit with a Cobas e411 analyzer (Roche Diagnostics, Basel, Switzerland) (IL-6 normal limit in healthy individuals <7 pg/mL).

### 2.4. Statistical Analyses

The chi-square test was applied to examine the frequency differences of categorical variables in different groups of patients. Given the non-normal distribution observed in variables, the Kruskal–Wallis test was used to identify statistical differences of continuous variables across more than two groups of patients. The nonparametric Mann–Whitney *U* test was used to analyze the distribution of IL-6 between two groups of patients. Correlations of IL-6 with other laboratory parameters were analyzed using Spearman's correlation test. All the statistical determinations were analyzed using SPSS (version 25) and/or GraphPad Prism v 9.0. The differences were considered statistically significant at *p* values of less than 0.05.

## 3. Results

A total of 86 patients diagnosed with SARS-CoV-2 infection from November 1, 2020, to January 31, 2021, were included in the study. Of the analyzed group, 73 patients (85%) were hospitalized while 14 were ambulatory patients. The median (IQR) age of patients was 61.50 (49.75–67.25) years with 50 male patients (58.1%) and 36 females (41.9%). The duration of symptoms prior to hospitalisation was 6 days (3.00–8.75), and the median (IQR) of hospitalisation days was 11.5 days (9.00–15.00). Most patients ranked in the “severe” category group (50 patients or 58.1%) whereas 23 patients (26.7%) were moderate. Pulmonary infiltrates were present in 65 (75.6%) of patients and 52 (60.5%) were in oxygen therapy. Sixty-one patients had other comorbidities (71%) whereas 43% of patients had hypertension. Of a total of 86 patients, 71 (82.6%) were treated with corticosteroids (dexamethasone or methylprednisolone) and 45 patients (52.3%) were treated with antivirals (favipiravir or remdesivir). Four patients in the critical group died, and so the overall mortality rate was 4.7% (Table 1).

### 3.1. Age, Corticosteroid, and Antibiotic Use Were Associated with Disease Severity

Most patients included in this study were in the severe group. There was no significant difference in disease severity by sex, whereas there was a significant difference between age groups. Although the highest number of patients with hypertension and diabetes were in the severe group, there were no statistically significant differences between groups. Statistically significant differences in disease severity were found between patients treated with corticosteroids and without corticosteroids and patients treated with and without antibiotics (Table 2).

When analyzing the frequency of biochemical and hematological parameters, we found that the levels of IL-6 were increased in 54 (62.8%) patients whereas CRP, ESR, and granulocyte percentages were increased in 57 (67.1%), 56 (83.6%), and 51 (68.9%) patients, respectively. Lymphocytes were decreased in 52 (68.4%) patients. More than 50% of patients had normal levels of D-dimer, LDH, CK, and WBC count (Table 3).

### 3.2. Several Inflammatory and Hematological Parameters Were Associated with Disease Severity

In this study, we sought to determine which inflammatory and hematological parameters are linked to worse clinical outcomes. The distribution of CRP, LDH, ESR, WBC, and percentages of granulocytes were significantly different across disease severity groups with higher values in more severe patients whereas lower percentages of lymphocytes and monocytes and platelet count in more severe patients. In contrast, the distribution of D-dimer and CK did not show significant differences across the different disease severity groups although levels were higher in severe and critically ill patients (Table 4).

### 3.3. Age and History of Hypertension Influence the IL-6 Levels in COVID-19 Patients

**Figure 1:**
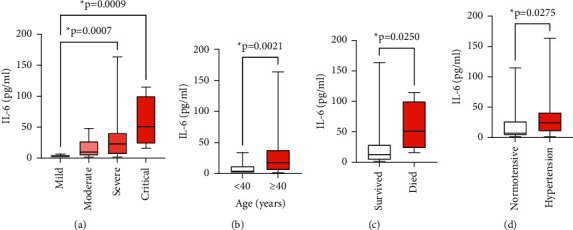
Distribution of IL-6 across the different groups of patients with SARS-CoV-2 infection.

Levels of IL-6 were significantly different between patients <40 years and >40 years, with mean ranks being higher in the group of patients aged >40 years (*p*=0.021). Also, levels of IL-6 were significantly different across disease severity groups, with higher values of IL-6 in severe and critical patients (*p* ≤ 0.001). Analyzing the distribution of IL-6 in different groups including (i) comorbidities and (ii) disease outcomes, a significant difference was found between hypertensive and nonhypertensive patients (*p*=0.027) and between those who survived and died (*p*=0.025) (Figure 1).

(a) Baseline levels of serum IL-6 were classified into four groups (mild, moderate, severe, and critical). (b) Comparison of mean ranks of IL-6 between patients <40 years and >40 years. (c) Differences in IL-6 between patients who survived and deceased patients. (d) Differences in levels between hypertensive and nonhypertensive patients. Data are from *n* = 86 patients, analyzed using the Kruskal–Wallis test for (a) and Mann–Whitney *U* test for (b), (c), and (d).

### 3.4. Correlations between IL-6 and Other Inflammatory and Hematological Parameters

**Figure 2:**
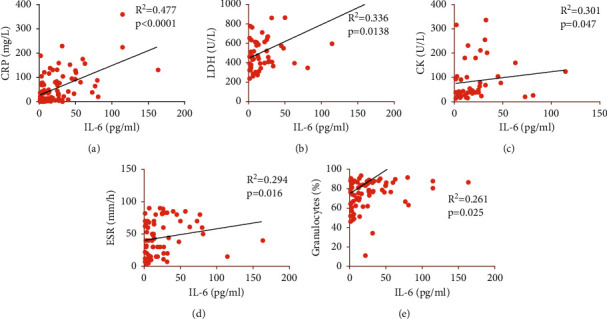
Correlation of IL-6 with other inflammatory and hematological parameters. Pearson correlation and linear regression were used for statistical analysis. IL-6, interleukin 6; CRP, C‐reactive protein; LDH, lactate dehydrogenase; CK, creatine kinase; ESR, erythrocyte sedimentation rate.

When analysing the correlation of IL-6 with several inflammatory and hematological parameters (CRP, LDH, CK, D-Dimer, ESR, WBC, percentage of granulocyte, lymphocytes, monocytes, and platelet count), significant weak to moderate correlation was found between IL-6 and CRP (*p* ≤ 0.001), LDH (*p*=0.016), CK (*p*=0.047), ESR (*p*=0.013), and percentages of granulocytes (*p*=0.025) (Figure 2).

## 4. Discussion

Kosovo is a small country with a weak health system but with the youngest population in Europe. Most patients with COVID-19 in Kosovo develop mild forms of the disease while 10–15% manifest severe and critical forms requiring hospitalisation. This study included 86 COVID-19 patients with symptoms ranging from mild to critical treated during a three-month period (November 2020- January 2021). Male patients dominated the cohort, as has been the case in several other published papers [15, 16]. In this study, the gender did not significantly influence disease severity which is in line with other papers [17, 18] but in contrast to some studies showing that disease severity of COVID-19 was associated with male sex [19, 20]. Sex-specific features of the innate and adaptive immune systems account for an advantage in the defence against COVID-19 in females [20], but the protective role was not shown in this study.

When comparing disease severity according to age, we found significant differences between disease severity and different age groups. The results of this study are in line with a great proportion of COVID-19 studies which concluded that age was the dominant risk factor contributing to severe disease and adverse outcomes [6, 20–24]. Age-related comorbidities are the leading reason for the increased mortality observed in older ages. Comorbidities, such as hypertension, diabetes mellitus, and cardiovascular disease also were found to be risk factors for severe forms of the disease [17, 21, 22]. In this study, almost 70% of patients had an underlying disease, with hypertension and diabetes being the most common. Zhau et al. found lower percentages of patients to have comorbidities (48%) with hypertension only in 30% of patients whereas diabetes in 18% of patients (the median age of patients in that study was lower than in this study). From a pooled analysis of hypertension in patients with severe or nonsevere COVID-19, or in COVID-19 survivors versus nonsurvivors, Lippi et al. found that hypertension was associated with up to 2.5-fold higher risk of severe form or fatal COVID-19, especially in older individuals [25]. It was hypothesised that hypertensive patients were more prone to SARS-CoV-2 infection due to the fact that they were under ACE inhibitors and angiotensin receptor blockers, which could enhance ACE2 expression, a linking SARS-CoV-2 molecule. Therefore, it is assumed that hypertension increases the risk of a worse prognosis of COVID-19. In this study, although the highest number of patients with hypertension and diabetes is in the severe group, there were no statistically significant disease group differences between hypertensive and nonhypertensive patients. There are other reports confirming this where they found no association between hypertension and disease severity [26, 27].

The distribution of CRP, LDH, and ESR and percentages of granulocytes were significantly different across disease severity groups with higher values in more severe patients whereas WBC count, percentages of lymphocytes and monocytes, and platelet count with lower values in more severe patients. Several studies have shown that exaggerated inflammatory responses and cytokine release syndrome (CRS) might be the main cause of COVID-19 pathogenesis, and thereby fatality [28–30]. In a meta-analysis of 40 studies conducted by Melo et al. (2021), it was concluded that elevated levels of IL-6, CRP, LDH, D-dimer, procalcitonin, aspartate aminotransferase, creatinine, leukocytes, and neutrophils with lymphopenia and thrombocytopenia are important biomarkers of CRS [31].

IL-6 is one of the most prominent proinflammatory cytokines. Normal physiological concentrations of IL-6 in human serum are relatively low (1–5 pg/ml), but these are rapidly elevated in disease settings. In COVID-19 patients, increased levels of IL-6 are recorded, especially in patients with a severe-to-critical form of the disease [18, 32], and an increasing mean in IL‐6 on admission was associated with an increased likelihood of mortality [12]. In this study, levels of IL-6 were elevated in 62.8% of patients with higher values of IL-6 in severe and critical patients who were also deceased. COVID-19 in elderly people is associated with high levels of proinflammatory cytokines [33] which play a critical role in the development of cytokine storm in severe forms of the disease [34]. In this study, we also found that levels of proinflammatory IL-6 cytokine were higher in the elderly. History of hypertension was found to be an independent risk factor for COVID-19-induced CRS [35]. In this study, higher levels of IL-6 were also seen in hypertensive patients compared to nonhypertensive patients.

Results of this study also show that levels of IL-6 significantly correlated with CRP, ESR, LDH, granulocytes, and biomarkers which were also associated with disease severity, a finding which opposes that by Santa Cruz et al. [8]. It is known that increased levels of IL-6 during inflammatory responses trigger the synthesis of acute-phase proteins such as CRP [36] which parallels the severity of inflammatory response [37]. An extensive body of literature shows that IL-6 modulates the innate immune system, including hematopoiesis [38]. Liu et al. found that exogenous IL-6 stimulated granulopoiesis *in vivo* in the absence of G-CSFR signals which indicates that IL-6 is an independent regulator of granulopoiesis [39]. Results from this study also show this association; a positive correlation between IL-6 and percentages of granulocytes was found. Leucopenia, lymphopenia, and neutrophilia are usually seen in viral infections [16, 40–42] and in COVID-19 [7, 43] which was also confirmed in this study. Neutrophilia indicates the intensity of inflammatory response, while lymphopenia suggests the damage to cells of the immune system [37].

This study has some limitations. The first one is the relatively small sample size, which may reduce the statistical power of the study. Another limitation is that due to the circumstances, it was not possible to have all the parameters measured for each patient. In this regard, the results and conclusions should be interpreted with caution.

## 5. Conclusion

In conclusion, inflammatory response with increased levels of IL-6 and other dysregulated hematological and inflammatory parameters may predict disease severity and outcome of the SARS-CoV-2 infection.

## Figures and Tables

**Table 1:** Demographic and clinical parameters of COVID-19 patients.

Parameters		Patients
Sex *N* (%)	Female	36 (41.9)
	Male	50 (58.1)
Age	Median (IQR)	62 (50–67)
Hospitalisation *N* (%)	Yes	73 (84.9)
Days of hospitalisation	Median (IQR)	11.50 (9.00–15.00)
Days prior to hospitalisation	Median (IQR)	6.00 (3.00–8.75)
Pulmonary infiltrates	Yes	65 (75.6)
Oxygen therapy	Yes	52 (60.5)
Disease severity *N* (%)	Mild	9 (10.5)
	Moderate	23 (26.7)
	Severe	50 (58.1)
	Critical	4 (4.7)
Medications used during illness *N* (%)	Corticosteroids	71 (82.6)
	Antivirals	45 (52.3)
	Antibiotics	82 (95.3)
Comorbidities *N* (%)	Hypertension	37 (43.0)
	Diabetes	15 (17.4)
	Cancer	7 (8.1)
	Hypothyroidism	2 (2.3)
Outcome *N* (%)	Recovery	82 (95.3)
	Death	4 (4.7)

**Table 2:** Distribution of demographic and clinical parameters across diseases severity groups.

		Mild	Moderate	Severe	Critical	*p* value
		9	23	50	4	
Sex	Male	3	12	32	3	0.286^a^
Age	Mean ranks	15.50	38.17	49.62	60.63	*0.001* ^b^
*Comorbidities*						
Hypertension	Yes	1	10	24	2	0.228^a^
Diabetes	Yes	0	1	13	1	0.062^a^
Cancer	Yes	0	4	2	1	0.107^a^
Hospitalisation	Yes	1	18	50	4	≤*0.001*^a^
*Medications used during illness*						
Corticosteroids	Yes	0	21	46	4	≤*0.001*^a^
Antivirals	Yes	2	13	27	3	0.230^a^
Antibiotics	Yes	7	23	50	2	≤*0.001*^a^

*p* values less than 0.05 are highlighted in italic. ^a^Pearson *χ*2 test. ^b^Kruskal–Wallis test.

**Table 3:** Frequencies of biochemical and hematological parameters in the whole group of patients.

	Reference value	Total	Low		Normal		Elevated	
			*N*	%	*N*	%	*N*	%
IL-6 (pg/ml)	<7	86	—	—	32	37.2	54	62.8
CRP (mg/L)	10	85	—	—	28	32.9	57	67.1
D-dimer (ng/ml)	500	80	—	—	46	57.5	34	42.5
LDH (U/L)	480	53	—	—	29	54.7	24	45.3
CK (U/L)	♂−171, ♀−147	44	—	—	36	81.8	8	18.2
ESR (mm/h)	♂−10, ♀−15	67	—	—	11	16.4	56	83.6
WBC (×10^9^/L)	4–10	84	13	15.5	43	51.2	28	33.3
Granulocytes (%)	60–72	74	10	13.5	13	17.6	51	68.9
Lymphocytes (%)	25–33	76	52	68.4	13	17.1	11	14.5
Monocytes (%)	3–7	63	21	33.3	30	47.6	12	19.0
Platelet count	100–400	83	3	3.6	78	94.0	2	2.4

IL-6, interleukin 6; CRP, C‐reactive protein; LDH, lactate dehydrogenase; CK, creatine kinase; ESR, erythrocyte sedimentation rate; WBC, white blood cells. For parameters which are expressed in normal and elevated values only, the respective cells in the low-value column are left blank.

**Table 4:** Distribution of biochemical and hematological parameters across disease severity groups.

		Mild	Moderate	Severe	Critical	*p* value
CRP (mg/L)	*n* (Mean rank)	8 (11.69)	23 (28.48)	50 (53.67)	4 (55.75)	*≤0.001*
D-dimer (ng/ml)	*n* (Mean rank)	8 (30.56)	20 (33.73)	48 (43.49)	4 (58.38)	0.097
LDH (U/L)	*n* (Mean rank)	0 (0.00)	16 (17.06)	33 (30.85)	4 (35.00)	*0.008*
CK (U/L)	*n* (Mean rank)	0 (0.00)	11 (17.91)	30 (23.32)	3 (31.17)	0.235
ESR (mm/h)	*n* (Mean rank)	4 (9.75)	21 (26.76)	38 (39.12)	4 (47.63)	*0.003*
WBC (×10^9^/L)	*n* (Mean rank)	8 (14.25)	22 (40.00)	50 (47.74)	4 (47.25)	*0.004*
Granulocytes (%)	*n* (Mean rank)	8 (12.13)	19 (25.24)	43 (46.90)	4 (45.50)	*≤0.001*
Lymphocytes (%)	*n* (Mean rank)	8 (62.19)	20 (50.58)	44 (29.41)	4 (30.75)	*≤0.001*
Monocytes (%)	*n* (Mean rank)	8 (50.44)	18 (39.08)	33 (24.91)	4 (21.75)	*0.001*
Platelet count	*n* (Mean rank)	8 (33.75)	22 (43.07)	49 (45.40)	4 (11.00)	*0.036*

*p* values less than 0.05 are highlighted in italics. Statistical analyses were performed with the Kruskal–Wallis test. IL-6, interleukin 6; CRP, C‐reactive protein; LDH, lactate dehydrogenase; CK, creatine kinase, ESR, erythrocyte sedimentation rate; WBC, white blood cells.

## Data Availability

The excel data used to support the findings of this study are available from the corresponding author upon request.
